# A Practical Clinical Approach to Navigate Pulmonary Embolism Management: A Primer and Narrative Review of the Evolving Landscape

**DOI:** 10.3390/jcm13247637

**Published:** 2024-12-15

**Authors:** Kevin Benavente, Bradley Fujiuchi, Hafeez Ul Hassan Virk, Pavan K. Kavali, Walter Ageno, Geoffrey D. Barnes, Marc Righini, Mahboob Alam, Rachel P. Rosovsky, Chayakrit Krittanawong

**Affiliations:** 1 Department of Medicine, John A. Burns School of Medicine, University of Hawai’i, Honolulu, HI 96813, USA; kevinb33@hawaii.edu (K.B.); blf@hawaii.edu (B.F.); 2 Harrington Heart & Vascular Institute, Case Western Reserve University, University Hospitals Cleveland Medical Center, Cleveland, OH 44106, USA; hafeezvirkmd@gmail.com; 3 Interventional Radiology, Mallinckrodt Institute of Radiology, Washington University School of Medicine in St. Louis, St. Louis, MO 63110, USA; pavan.kavali@wustl.edu; 4 Department of Clinical Medicine, University of Insubria, 21100 Varese, Italy; walter.ageno@uninsubria.it; 5 Frankel Cardiovascular Center, Department of Internal Medicine, University of Michigan Medical School, Ann Arbor, MI 48109, USA; gbarnes@med.umich.edu; 6 Division of Angiology and Hemostasis, Department of Medicine, Geneva University Hospitals and Faculty of Medicine, CH-1211 Geneva, Switzerland; marc.righini@hug.ch; 7 The Texas Heart Institute, Baylor College of Medicine, Houston, TX 77030, USA; mahboob.alam@bcm.edu; 8 Division of Hematology & Oncology, Department of Medicine, Massachusetts Hospital, Boston, MA 02114, USA; rprosovsky@mgh.harvard.edu; 9 Section of Cardiology, Cardiology Division, NYU Langone Health and NYU School of Medicine, 550 First Avenue, New York, NY 10016, USA

**Keywords:** PE, pulmonary embolism, VTE

## Abstract

Advances in imaging, pharmacological, and procedural technologies have rapidly expanded the care of pulmonary embolism. Earlier, more accurate identification and quantification has enhanced risk stratification across the spectrum of the disease process, with a number of clinical tools available to prognosticate outcomes and guide treatment. Direct oral anticoagulants have enabled a consistent and more convenient long-term therapeutic option, with a greater shift toward outpatient treatment for a select group of low-risk patients. The array of catheter-directed therapies now available has contributed to a more versatile and nuanced armamentarium of treatment options, including ultrasound-facilitated thrombolysis and mechanical thrombectomy. Research into supportive care for pulmonary embolism have explored the optimal use of vasopressors and volume resuscitation, as well as utilization of various devices, including right ventricular mechanical support and extracorporeal membrane oxygenation. Even in the realm of surgery, outcomes have steadily improved in experienced centers. This rapid expansion in diagnostic and therapeutic data has necessitated implementation of pulmonary embolism response teams to better interpret the available evidence, manage the utilization of advanced therapies, and coordinate multidisciplinary care. We provide a narrative review of the risk stratification and management of pulmonary embolism, with a focus on structuralizing the multidisciplinary approach and organizing the literature on new and emerging therapies.

## 1. Introduction

Pulmonary embolism (PE) continues to contribute to significant morbidity and mortality in patients worldwide, despite large advances in treatment. Patients presenting with hemodynamic collapse demonstrate mortality rates between 17.2% and 42.1%, even with utilization of advanced support such as extracorporeal membrane oxygenation (ECMO) [1,2]. Amongst all comers with PE, contemporary data for 2016–2019 demonstrate an in-hospital mortality of 6.5% [1]. Innovations in the treatment of PE have rapidly expanded to include a variety of surgical, mechanical, catheter-based, and medical therapies. In high-risk PE patients reported from a large nationwide database, catheter-directed therapies were used in 6.6% of cases [3]. However, the strength of evidence across the diverse menu of therapeutic modalities is heterogeneous, with guideline recommendations that either insufficiently guide management or differ considerably among societies. Despite the widespread adoption of multidisciplinary pulmonary embolism response teams (PERT), originally aimed at navigating treatment within this relatively evidence-poor field, as well as several ongoing trials to decipher the optimal treatment modalities for PE at various levels of risk, the overwhelming absence of level one evidence makes management of high-risk PE a complex and nuanced topic. Here, we review the pathophysiology, risk-stratification, interdisciplinary care, and evidence regarding therapies in the management of PE in the context of societal guidelines.

## 2. Pathophysiology of Acute PE

### 2.1. Pathogenesis

Acute pulmonary emboli are largely a consequence of deep venous thrombosis of the lower extremities, most frequently of the calf and femoropopliteal veins, which dislodge and embolize to the pulmonary arteries. Such phenomena have traditionally been attributed to a combination of elements of Virchow’s triad, including venous stasis, endothelial injury, or hypercoagulable state. As a result, many conditions may predispose an individual to developing deep vein thrombosis and subsequent PE, and risk factors may be categorized on whether they were inherited or acquired. Acquired risk factors include recent trauma, surgery, immobility, malignancy, and pregnancy or estrogen therapy, with inherited risk factors including conditions such as factor V Leiden, protein C or S deficiencies, or mutations in antithrombin or prothrombin [4].

### 2.2. Pulmonary Effects

The hemodynamic effects of PE are attributable first at the level of the pulmonary vasculature. In patients without prior cardiovascular disease, the degree of mechanical obstruction and clot burden is poorly correlated with hemodynamic compromise [5]. Even partial mechanical occlusion of the pulmonary artery as small as 30% may lead to consistent increases in pulmonary artery pressure, and can be better explained by compensatory thromboxane A_2_- and serotonin-mediated vasoconstriction in a physiologic attempt to maintain pulmonary perfusion [6]. Ultimately, impaired blood flow to the corresponding alveolar tributaries contributes to ventilation–perfusion mismatching and shunting of oxygen-deprived venous blood into arterial circulation. Patients can develop hypoxia recalcitrant to supplemental oxygen, further worsening pulmonary vasoconstriction.

### 2.3. Cardiovascular Effects

Downstream, the increase in pulmonary vascular resistance challenges the right ventricle to increase stroke volume via catecholamine- and neurohormone-induced chronotropy and inotropy. Because of this mechanism, , some patients may only display moderate elevations in PA pressure, even with high degrees of mechanical obstruction. However, the non-preconditioned right ventricle is unable to effectively overcome acute increases in pulmonary artery pressures of more than 40 mmHg, with the magnitude of pulmonary artery pressures seen in PE often exceeding four to five times normal values [6,7]. Excess increased end-diastolic volume and distention further compromises effective RV augmentation of stroke volume via the Frank–Starling mechanism, with subsequent excretion of brain natriuretic peptide (BNP). As the RV becomes distended, the duration of RV contraction lengthens relative to LV contraction, leading to dyssynchrony. RV pressure overload causes the interventricular septum to flatten, or even paradoxically bow leftward, impairing LV filling and decreasing stroke volume without decreasing ejection fraction [8]. RV ischemia ensues, causing a leak of cardiac troponin, further reducing RV systolic function and transpulmonary flow and contributing to hypoperfusion and cardiogenic shock. These changes may be magnified in patients with pre-existing cardiopulmonary comorbidities, further contributing to hastened decline. Thus, numerous therapies have been tested and directed at relieving both the mechanical and physiologic obstruction, offloading the right ventricle, and improving stroke volume in PE patients. As patents spiral further into hemodynamic collapse, the interplay of vascular obstruction, coagulopathy, respiratory insufficiency, and cardiac failure is amplified. The complex pathophysiology of the disease, as well as the therapies designed to treat it, transverse the domain of multiple specialties, necessitating multidisciplinary teams composed of cardiologists, vascular surgeons, pulmonary intensivists, interventional radiologists, and hematologists to collaborate not only in the care of individual patients but also in the drafting of clinical practice guidelines.

## 3. Risk Stratification

### 3.1. Historical Perspective

The treatment of PE is dependent on risk stratification, with the American Heart Association (AHA) and European Society of Cardiology (ESC) both adopting similar methods of classifying patients. Major factors including hemodynamic stability, RV function, myocardial injury, and clinical risk factors. The initial evaluation begins with an assessment of circulatory status, where patients presenting with obstructive shock (systolic blood pressure of <90 mmHg or ≥90 mmHg requiring vasopressor support combined with hypoperfusion in a patient without hypovolemia, sepsis, or new arrhythmias), persistent hypotension (SBP < 90 mmHg or >40 mmHg drop persisting >15 min), or cardiac arrest are classified as having a high-risk pulmonary embolism (ESC) or massive pulmonary embolism (AHA) [9,10]. These criteria come from registry data demonstrating in-hospital mortality of 15% in patients presenting with SBP <90 mmHg and mortality of 65% in patients presenting with cardiac arrest [11]. Patients without hypotension who have evidence of either RV strain/dysfunction by imaging or RV injury/ischemia by serum biomarkers are considered intermediate risk (ESC) or submassive (AHA). Accounting for 35–55% of patients hospitalized with PE, the intermediate-risk/submassive group is both large and heterogeneous. Some patients remain stable despite elevations in RV/LV ratio and biomarkers; however, others may quickly deteriorate to instability despite being considered intermediate risk.

To better classify these patients, the ESC makes the additional distinctions of intermediate high and intermediate low risk. If both myocardial injury and RV dysfunction are present, patients are classified in the intermediate high-risk group. If only one of the two is present, patients are regarded as intermediate low risk. Despite these additional subclasses, the intermediate high-risk group remains still a challenging population, where accurately predicting which patients are likely to deteriorate and may benefit from advanced therapies is still challenging. Patients who do not meet criteria for any of these classes are considered low risk by both guidelines.

### 3.2. Clinical Prognostic Tools

While a patient’s history, vital signs, laboratory results, and imaging findings are required to optimize prognostication, several tools have been developed to assist with assigning risk. For patients managed in the inpatient setting, the ESC recommends applying the Pulmonary Embolism Severity Index (PESI) score or its simplified version (sPESI), given its excellent sensitivity (88% and 92%, respectively) and extensive validation [12]. A PESI score < 86 or sPESI < 1 indicates low risk, while higher scores stratify patients into at least intermediate-risk. Alternatively, the Hestia tool, using a cutoff score of <1, can help differentiate patients who may be at low mortality risk and are suitable for treatment at home. Hestia is a noninferior tool to the sPESI, with similar rates of estimating mortality, bleeding, and recurrent VTE [13]. All these scores take into consideration hemodynamic stability and incorporate risk factors from a patient’s medical history. They do not incorporate an evaluation of RV function or cardiac damage, which are often assessed simultaneously and offer additional prognostic value. Thus, these scores must be applied only in the context of cardiac biomarkers and imaging. In the absence of hemodynamic compromise, RV dysfunction, or myocardial injury by biomarkers, however, the ESC considers an isolated positive sPESI or PESI score as fulfilling criteria for intermediate low-risk PE. Similarly, if the sPESI, PESI, or Hestia score is negative and there is no hemodynamic compromise, the isolated presence of RV dysfunction or biomarker evidence of myocardial injury is sufficient for intermediate-risk PE, but not low-risk PE.

Another risk stratification system, the Bova score, goes further in incorporating both cardiac troponin and either trans-thoracic echocardiogram (TTE) or computed tomography angiogram (CTA) evidence of RV dysfunction [14], although it is a system that has not been used as a method to guide therapeutic interventions in clinical trials. Amongst scores validated in intermediate-risk patients, the FAST score and the Composite Pulmonary Embolism Shock (CPES) score may be helpful to further prognosticate outcomes amongst this heterogeneous population and select patients who might preemptively benefit from advanced therapies. While both scores utilize tachycardia and troponin as components, the FAST score additionally takes into account the presence of syncope, while the CPES score includes RV dysfunction, elevated BNP, residual deep vein thrombosis (DVT), and central location of PE as considerations [15,16]. Direct comparisons of the accuracy of these tools offer conflicting results in terms of a clearly superior scoring system [17,18]. However, the simplicity of these tools allows for wide adoption and utilization, especially amongst frontline providers in emergency medicine and internal medicine, such that prompt triaging and specialist consultation can be obtained if necessary (Table 1).

## 4. Cardiac Evaluation

In hemodynamically stable patients, an assessment of RV function and myocardial injury should nevertheless be made, regardless of the results of risk stratification tools. Findings of RV dysfunction are associated with increased mortality even in patients classified as low risk by validated scoring systems [19]. Furthermore, up to 31% of normotensive patients with PE are found to have echocardiographic evidence of RV dysfunction, with 10% of these patients going on to develop latent hemodynamic compromise [20]. Formal evaluation of RV function can be conducted via laboratory assessment of both troponin and BNP, as well as imaging techniques such as TTE or CTA. An RV/LV ratio >1 has been associated with 2.5 times higher all-cause mortality and 5 times higher rates of PE-related mortality [21]. Additionally, high degrees of contrast refluxing to the inferior vena cava (IVC) on CTA signify RV dysfunction and have been linked to higher rates of 30-day mortality or clinical deterioration (specificity up to 98%) [22]. Troponin elevation is closely correlated with RV dysfunction in patients presenting with PE; however, it also signifies myocardial injury to the bilateral chambers of the heart [23]. Compared to those with normal levels, elevated BNP also closely correlates with RV dysfunction and is associated with in-hospital or 30-day mortality of 14% (OR 6.5) and risk of adverse outcomes in 32% of patients (OR 6.3) [24]. Overall, an elevated troponin is most significantly associated with early mortality (OR 6.25), followed by echocardiographic RV dysfunction (OR 4.19), and then by elevated BNP (OR 3.71) [19]. Early echocardiography is crucial in the evaluation of hemodynamically unstable patients; however, RV dysfunction may be challenging to interpret, leading to a delayed or missed diagnosis [25]. Thus, early consultation with cardiology and radiology can help more promptly identify RV failure and provide assistance in differentiating between non-PE causes of RV dysfunction, such as right heart infarctions. Combined RV dysfunction and myocardial injury, seen in the intermediate high-risk class, has higher 30-day mortality (up to 38%) compared to either an elevated troponin alone or RV dilation alone (23% and 9%, respectively) [26].

## 5. Treatment of Acute PE Based on Risk Category: Low-Risk PE

### 5.1. Anticoagulation in Low-Risk PE

For those with low-risk PE, the standard of care is anticoagulation, which can be initiated in the emergency department. The recommended type of maintenance anticoagulation used in stable patients with pulmonary embolism following acute evaluation and stabilization has shifted away from vitamin K antagonists and toward direct oral anticoagulants (DOACs). The EINSTEIN trial was the first study to suggest noninferiority of DOACs compared to warfarin in patients with PE, with similar rates of recurrent symptomatic venous thromboembolism and lower rates of bleeding demonstrated with rivaroxaban [27]. The AMPLIFY trial replicated these results with the twice-daily DOAC apixaban, demonstrating noninferiority to warfarin in preventing the combined primary outcome of recurrent VTE or death from VTE [28]. Akin to these studies, the Hokusai-VTE and RE-COVER trials also found noninferiority of edoxaban and dabigatran to warfarin, respectively, further solidifying the efficacy of DOACs as a likely class effect [29,30].

Both the EINSTEIN and AMPLIFY trials included a subgroup of patients who did not receive initial anticoagulation with a parenteral agent. Instead, the very first medication initiated for anticoagulation was a DOAC. This supports the approach of utilizing DOACs as both the initial and the long-term choice of anticoagulant over initial treatment with a parenteral agent. This strategy was further supported by the HOT-PE trial, which enrolled 525 patients with low-risk PE to undergo early discharge with home rivaroxaban therapy. Despite single-agent DOAC therapy being prescribed as the initial and only type of anticoagulation in 22.7% of patients, symptomatic VTE occurred in only 2.1% of patients overall. These data support single-agent DOAC use to help facilitate early discharge from the hospital, eliminating the need for parenteral or bridging anticoagulation [31].

### 5.2. Outpatient Management

If the patient has no other indications for admission, has adequate family/social support, and can readily access medical care as needed, they can be safely discharged home to continue their anticoagulation. The Hestia study was the first to validate this approach, demonstrating a low 3-month incidence of VTE recurrence (2.0%) or death (1.0%) amongst 297 patients not meeting the Hestia criteria [32]. An international randomized controlled trial involving 399 patients further supported this approach as being noninferior to hospital admission. There was no significant difference in regard to recurrent VTE or mortality at 3 months, specifically for low-risk patients delineated by PESI criteria class I or II [33]. However, an assessment of RV function is critical prior to consideration of discharge.

While both the Hestia and PESI criteria help to stratify risk in terms of the likelihood of patients experiencing fatal events, the Hestia criteria further include exclusion of social determinants that may hinder favorable outcomes. Thus, a hybrid approach that includes both risk stratification with a tool such as the PESI or SPESI and consideration for social determinants of health has been recommended [9]. Despite the proven safety and efficacy of this approach in low-risk PE, adoption has been abysmal in the US, with only 4.1% of newly diagnosed PE patients discharged home without admission in one 61,070-patient cohort between 2016 and 2018. This occurs despite an estimated 30–51% of pulmonary embolism cases being suitable for management at home [34]. If an outpatient approach is pursued, coordination and assurance of outpatient follow-up plays a vital role in monitoring for complications and recurrence of PE. In patients with symptomatic PE, recurrence rates are reported at up to 19% at 24 months, and while rare, 0.6% of patients will go on to develop chronic thromboembolism pulmonary hypertension (CTEPH) [35,36]. Thus, these patients may require additional reimaging if either is suspected.

### 5.3. Coordination of Care

Patients with low-risk PE are increasingly being managed exclusively by their primary care provider and initiated on DOAC treatment or referred first to the emergency department from the clinic, only to be discharged directly back home for outpatient management [28]. Thus, increased collaboration and education between pulmonary, cardiology, vascular, and hematology specialists with family medicine and internal medicine practitioners will likely be beneficial to ensure that appropriate care and monitoring is being administered for these patients. Early referral to a hematologist to define the etiology and duration of long-term anticoagulation, particularly in cases where malignancy is suspected or an incidental PE is discovered, may also be helpful [37,38].

For patients with limited access to care, involvement of case management and pharmacy may be warranted to help facilitate obtaining medications and follow-up. In this regard, institutionally implemented protocols may serve to streamline treatment and transition care from the emergency room to an outpatient setting. Kabrhel et al. found that introduction of a PE treatment protocol resulted in a higher rate of low-risk PE being managed in the outpatient setting (28% from 18%, *p* < 0.001) [39,40]. Additionally, the American College of Emergency Physicians (ACEP) point-of-care toolkit provides a concise, yet comprehensive guide for the diagnosis, risk stratification, management, and follow-up of low-risk PE [41].

## 6. Intermediate Low-Risk PE

### 6.1. Anticoagulation in Intermediate Low-Risk PE

Patients with concerning clinical risk factors, indicated by a positive validated risk-assessment score such as the sPESI, should be categorized as having at least intermediate low-risk pulmonary embolism, even in the absence of RV dysfunction or myocardial injury, and promptly hospitalized. This approach is supported by validation of the sPESI tool, in which a score of 0 vs. ≥1 was associated with increased mortality (0.5% vs. 8.1% respectively) [42]. Anticoagulation is the mainstay of therapy and recommended as a class Ic recommendation to be initiated even prior to the result of confirmatory diagnostic tests if there is suspicion of at least an intermediate to high risk of PE. Similar to low-risk patients, intermediate-risk patients may be started directly on oral DOACs employing a single-agent anticoagulation strategy [43]. If a parenteral agent is felt necessary, low-molecular-weight heparin (LMWH) is preferred over unfractionated heparin (UH), even in cases where further intervention is planned. This recommendation originates from multiple trials, including a large meta-analysis demonstrating a trend toward superior efficacy in preventing recurrent venous thromboembolism, as well as a decreased rate of bleeding in patients receiving LMWH compared to UFH [44].

### 6.2. Catheter-Directed Therapies in Intermediate Low-Risk PE

The use of catheter-directed therapies and fibrinolytics has not been adequately studied in this population due to the perceived risk of bleeding outweighing the potential benefits. However, results extracted from small subgroup analyses have yielded promising results for mechanical thrombectomy (MT), catheter-directed thrombolysis (CDT), and hybrid catheters. 

Interim data from the FLASH registry, a multicenter collaboration compiling data from patients undergoing percutaneous MT with the FlowTriever system, revealed a small number of intermediate low-risk patients who received the intervention [45]. Of these patients who underwent percutaneous thrombectomy (36 patients, 7.2%), there was a statistically significant reduction of 27% in pulmonary artery pressure after MT [46].

Data on the hemodynamic effects of CDT via the EKOS catheter also included a limited number of patients who were intermediate low risk. The EKOS catheter utilizes ultrasound energy to facilitate the action of chemical thrombolytics infused directly at the site of thrombus. The SUNSET trial, which demonstrated significant improvements in RV/LV diameter ratio with EKOS catheter-directed thrombolysis, included 5% of patients who were intermediate low risk [47].

For hybrid catheters, the RESCUE trial demonstrated the efficacy of the Bashir system in intermediate-risk patients with evidence of right heart strain on CT imaging. However, 8.3% of these patients had negative troponin/BNP biomarkers, thus representing an intermediate low risk subgroup [48]. The Bashir system deploys a basket-shaped mesh with multiple infusion ports distributed throughout the frame, enabling a hybrid of both mechanical thrombectomy and CDT, While these studies provide subgroups of interest, overall, there are still insufficient data to support any first-line therapy other than anticoagulation for intermediate low-risk PE patients currently, particularly given the high efficacy and safety with this approach.

## 7. Intermediate High-Risk PE

### 7.1. Anticoagulation in Intermediate High-Risk PE

Patients who have a combination of biomarker-confirmed myocardial injury, RV strain on imaging, and further supported by a positive PESI or sPESI, but who remain hemodynamically stable without hypotension are characterized as having intermediate high-risk PE. Management begins with LMWH as first-line anticoagulation unless contraindicated. Multiple trials, most notably the THESEE trial, have demonstrated noninferiority of LMWH compared to unfractionated heparin in the composite outcome of recurrent VTE, major bleeding, or death at both 8 and 90 days. This strategy further obviates some of the frequent monitoring and blood draws that occupy nursing and laboratory staff when unfractionated heparin therapy is employed [49]. If hemodynamic stability is maintained, anticoagulation with LMWH should be continued and the patient monitored typically for 2–3 days prior to transitioning to an oral agent.

**Figure 1:**
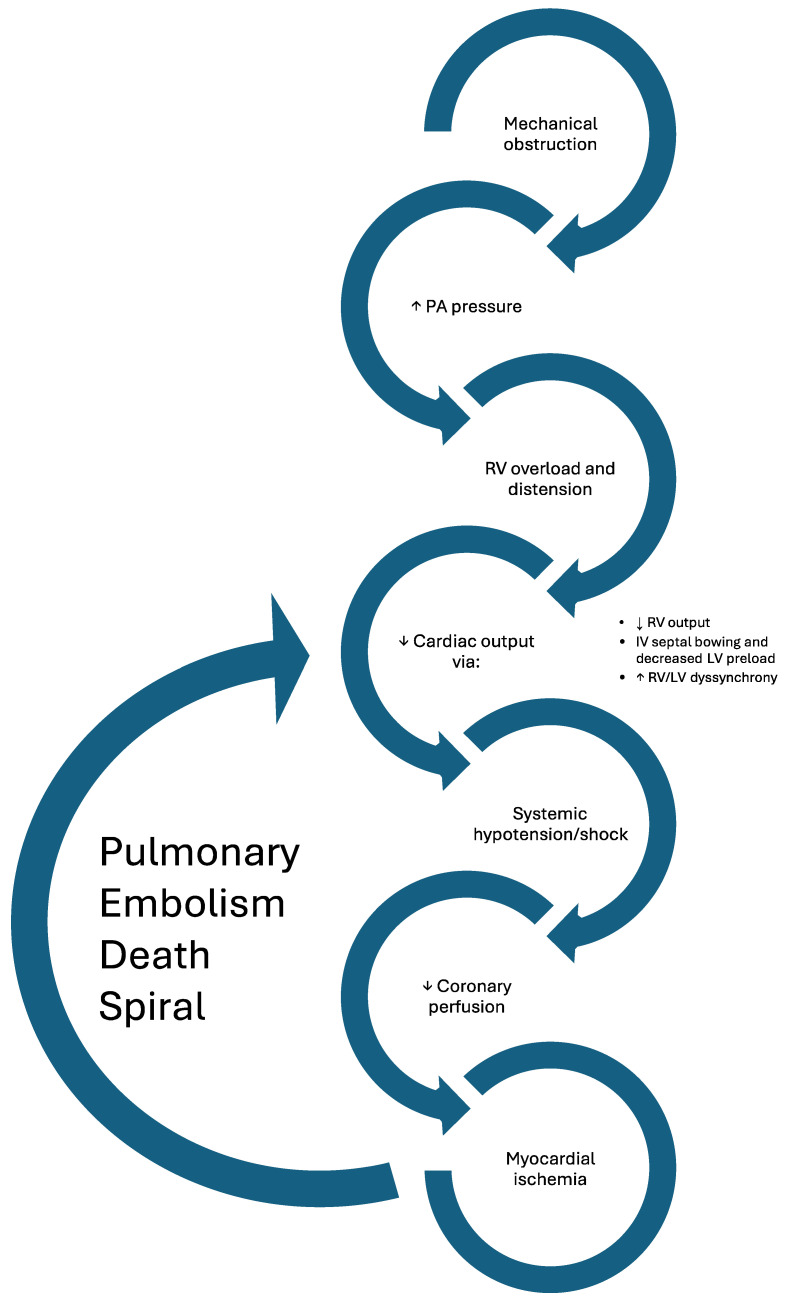
Representation of the cyclic hemodynamic effects of acute pulmonary embolism.

While some can be treated with anticoagulation alone, there is significant heterogeneity in the response to initial treatment within this patient population, and many progress to clinical instability. The Bova score can help further delineate risk and guide the decision to pursue advanced therapy amongst patients already classified in the intermediate high category [14], with patients who score 6 or greater, classified as Bova stage III, demonstrating a rate of death, hemodynamic collapse, or recurrent nonfatal PE of 42%. In patients at high risk of clinical deterioration, the indications, timing, and selection of advanced therapies are still poorly defined and continue to evolve. Here, we discuss treatment options for intermediate high-risk patients, including systemic thrombolysis, catheter-directed thrombolysis, and mechanical thrombectomy, with Figure 1 summarizing indications, available evidence, and clinical considerations for each.

### 7.2. Thrombolysis in Intermediate High-Risk PE

Early evidence for systemic thrombolysis in intermediate-risk PE initially comes in part from the small PAIMS2 study, which compared treatment of 20 patients with 100 mg of alteplase (10 mg bolus followed by 90 mg over 2 h) followed by continuous intravenous heparin compared to intravenous heparin alone, included 3 patients without hypotension [50]. Improvement in pulmonary artery obstruction as per the Miller index was significantly greater in the thrombolytic group, along with a decrease in mean pulmonary artery pressure. Another small trial by Goldhaber et al. randomized 46 hemodynamically stable patients to 100 mg of alteplase followed by heparin compared to heparin alone and found significant improvements in echocardiographic RV function and pulmonary perfusion imaging [51]. While these trials found improvements in hemodynamic profiles with the use of thrombolysis, they were unable to show that these changes correlated with improved clinical outcomes. Furthermore, bleeding occurred in 70% of patients in the PAIMS2 study, with major bleeding occurring in 28% in the trial by Goldhaber.

The PEITHO trial included 1006 patients and was the largest study to compare clinical outcomes in intermediate high-risk PE treated with either thrombolytics or anticoagulation. Patients included were hemodynamically stable with signs of RV dysfunction and myocardial injury and randomized to receive either IV tenecteplase followed by heparin or heparin alone. While the primary outcome of 7-day mortality or clinical deterioration was significantly improved with tenecteplase by 56%, it was driven by a large decrease in the rate of clinical deterioration, without significantly different 7-day mortality between the two groups (1.2% in the intervention group vs. 1.8% in the heparin-alone group). Of the additional secondary outcomes assessed, there was no difference in 30-day mortality; however, the thrombolysis group experienced significantly higher rates of major bleeding (6.3% vs. 1.2%) and hemorrhagic stroke (2% vs. 0.2%) [52]. While the PEITHO trial saw nonsignificant trends toward improved mortality at both 7 and 30 days, two meta-analyses that included intermediate-risk PE treated with thrombolytics had conflicting results regarding mortality benefit [53,54]. Because of the increased rates of bleeding complications (OR 5.55) without a clear benefit to mortality at the RCT level, the ESC recommends resorting to thrombolytic therapy only in intermediate high-risk patients who subsequently develop hemodynamic instability [9]. In an attempt to potentially mitigate the excess bleeding risk conferred by full-dose treatment, the PEITHO-3 trial is a randomized controlled trial currently in its recruitment phase that plans to assess 30-day mortality and bleeding risk for intermediate high-risk patients treated with half-dose alteplase [55].

Given that the mean time to hemodynamic collapse demonstrated in PEITHO was 1.79 days from randomization, the threshold to initiate treatment, particularly in Bova stage III patients in the intermediate high-risk category, should be supported by frequent monitoring and clinical reassessments for at least 48 h, and careful consideration that any potential benefits of this treatment come at the expense of increased frequency of major and intracranial bleeding [56]. Frequent collaboration between internal medicine and critical care specialists should be maintained to help identify patients who require early upgrading to a higher level of care for increased monitoring, allow prompt administration of thrombolysis in the appropriate setting, and provide diligent monitoring for hemorrhagic sequelae following thrombolysis.

### 7.3. Catheter-Directed Thrombolysis (CDT) in Intermediate High-Risk PE

While the use of systemic thrombolysis in intermediate high-risk patients with acute PE was shown to reduce hemodynamic decompensation, the high bleeding risk makes this an undesirable therapy for most patients. CDT offers a theoretical benefit of directly delivering thrombolysis to the site of the PE at lower doses, which may reduce the overall risk of bleeding while still offering benefit against hemodynamic decompensation. However, currently there are no published head-to-head studies comparing CDT to anticoagulation evaluating clinical outcomes such as mortality, hospital readmissions, or VTE recurrence. Instead, studies have focused on surrogate radiologic outcomes or have relied on single-arm designs without an anticoagulation comparison arm.

The ULTIMA trial evaluated the use of ultrasound-assisted catheter-directed thrombolysis in 59 intermediate high-risk PE patients using the EkoSonic endovascular system (EKOS catheter) [57]. The intervention resulted in a 0.30 (22%) mean decrease in RV/LV ratio at 24 h with no major bleeding, while the anticoagulation group exhibited no significant difference pre- and posttreatment. In addition, the authors demonstrated improvements in pulmonary artery pressures, right atrial pressure, and cardiac index.

Subsequently, the larger, single-arm SEATLE II study included 150 patients, 79.3% (*n* = 119) of which were patients with intermediate-risk PE, and extended the monitoring period for the primary outcome of change in the RV/LV ratio out to 48 h. There was a mean decrease of 0.42 (27%) in the RV/LV ratio at 48 h with a <1% rate of severe bleeding following receipt of CDT [58].

Finally, the CANARY trial included 94 patients and attempted to evaluate outcomes of ultrasound-guided CDT compared to IV thrombolysis, further extending the primary outcome out to 3 months in intermediate high-risk patients with PE. The trial was terminated early due to the COVID-19 pandemic, enrolling only 65% of the initially planned 144-patient cohort. The primary endpoint of 3-month echocardiographic RV/LV ratio greater than 0.9 was not achieved, but was numerically lower in the underpowered intervention arm (4.3% in the vs. 12.8% in the control). A composite of 3-month mortality and RV/LV ratio greater than 0.9 was observed in 4.3% of the intervention arm and 17.3% in the control arm (*p* = 0.048) [59].

Even in hybrid MT–CDT devices, radiologic evidence of improvements have been demonstrated. In the single-arm RESCUE trial, 109 patients treated with the Bashir catheter demonstrated a similar 0.56 (33%) decrease in RV/LV ratio diameter at 48 h with <1% rate of major bleeding [48].

To reconcile the accumulating body of evidence for CDT, several meta-analyses have evaluated both randomized control trials (RCTs) and observational data to determine its efficacy and safety. Overall, these studies found significantly lower 30-day, 90-day and in-hospital mortality for CDT compared to systemic anticoagulation, without significant differences in rates of bleeding complications. Although both studies concluded that catheter-directed thrombolysis should be considered a potential first line therapy in the treatment of intermediate-risk PE, they were observational in nature, and definitive large RCT-level datasets continue to be lacking [60,61]. Thus, in the absence of high-quality evidence, a discussion with a multidisciplinary team of cardiologists, pulmonologists, and critical care intensivists, who can be consulted to risk-stratify patients and further advise on deployment of an interventional radiologist/cardiologist with expertise in CDT, is often necessary.

The HI-PEITHO trial (CDT04790370) is an ongoing, multicenter, randomized trial comparing CDT with the EKOS system to anticoagulation alone in patients with intermediate high-risk PE and additional risk factors (e.g., tachycardia, relative hypotension, respiratory compromise) that has completed enrollment of 406 patients. Key outcomes for this study include mortality, recurrence of acute PE, and hemodynamic decompensation; however, with the current data available, anticoagulation remains the standard for intermediate high-risk patients [62].

### 7.4. Mechanical Thrombectomy in Intermediate High-Risk PE

Catheter-based suction and MT is an alternative approach to CDT that can be deployed with or without the use of thrombolytics. This allows for a potentially more immediate intervention, as it does not require hours of thrombolytic infusion, and theoretically further reduces any bleeding risk.

The EXTRACT-PE study enrolled 119 patients with intermediate high-risk PE to undergo suction thrombectomy with the Indigo aspiration catheter, resulting in a mean RV/LV diameter ratio reduction of 0.43 (27.3%), mean ICU stay of 1 day, 30-day all-cause mortality of 2.5%, and a 1.7% rate of major bleeding [63]. The Indigo device utilizes continuous suction produced by an electronic pump as the primary mechanism of clot removal.

The FLARE study produced similar results, enrolling 106 intermediate-risk PE patients to undergo catheter-directed mechanical thrombectomy with the FlowTriever system. In comparison to the Indigo device, the mechanism of FlowTriever utilizes a dual action: displacing clots via deployment of three expandable mesh disks along the length of the clot, and suction produced by a syringe and plunger at the operator end of the device. In the FLARE study, the RV/LV ratio was reduced by 0.38 (25.1%) with a 30-day all-cause mortality of 1% and 1% rate of major bleeding. Although technically designed as a single-arm study, when compared to a prespecified “context arm,” which enrolled patients in parallel treatment with non-FlowTriever therapies, in-hospital mortality was 1.9% in the mechanical thrombectomy group compared to 29.5% in the context arm [64]. However, it is important to note that this was not a randomized trial and there were key differences in patient selection for each of these two treatment arms.

The FLASH registry continued collecting data on the use of mechanical thrombectomy with the FlowTriever system, prospectively following mostly intermediate-risk PE patients. Interim results of 799 patients demonstrated a 6-month mortality of 4.6%, RV/LV ratio reduction of 0.43 (35.0%), prevalence of CTEPH in 1.0%, prevalence of CTED/post-PE syndrome of 1.9%, and normal RV function on echocardiogram in 95.1% of patients following mechanical thrombectomy [65]. There was no comparator arm in this registry study.

Hoping to address this, the PEERLESS II study is a multisite randomized trial comparing mechanical thrombectomy versus anticoagulation alone in patients with intermediate high-risk acute PE and additional risk factors (e.g., tachycardia, relative hypotension). Key outcomes include mortality, hemodynamic decompensation, readmission, and dyspnea scores [66]. Therefore, while mortality and bleeding rates for MT are promising thus far, superiority to anticoagulation has yet to be demonstrated in the RCT setting.

### 7.5. CDT vs. MT in Intermediate High-Risk PE

The optimal strategy CDT and MT is yet to be determined, and until recently, there had not been any completed RCTs to date. Several retrospective studies have attempted to elucidate the comparative efficacy of these therapies. One observational study included 458-patients treated between 2014 and 2021 and found no difference in a composite outcome of in-hospital mortality, significant bleeding, vascular complications, and need for mechanical support (12% in the CDT arm vs. 11% in the MT arm) [67]. This study was replicated in a retrospective cohort of 147 patients with intermediate- and high-risk PE, demonstrating no significant difference in mortality or similar safety in terms of procedure-related bleeding [68]. A meta-analysis in 2023 of 1403 patients from eight observational studies demonstrated no significant differences in mortality or bleeding between those patients who received CDT vs. MT [69].

The first RCT comparing the efficacy of catheter-based therapies, PEERLESS, randomized 550 patients with intermediate high-risk PE to either MT with the Inari FlowTriever or any CDT device at the discretion of the local PERT. Utilizing a win ratio to evaluate the composite hierarchical outcome of all-cause mortality, intracranial bleeding, major bleeding, clinical deterioration and/or bailout, or postprocedural ICU utilization, a win ratio of 5.01 was observed in favor of MT. The benefit was driven by significantly lower rates of clinical deterioration/bailout and reductions in both ICU admissions and length of stay, while the rates of intracranial hemorrhage and major bleeding were not significantly different. Furthermore, both hospital length of stay and rates of hospital readmission were significantly lower with MT. The PEERLESS trial represents huge progress in PE research, demonstrating the superiority of one catheter-based modality over another in a field where prior studies lacked patient-oriented clinical outcomes and appropriate comparator groups [70].

The ongoing PEERLESS2 trial (NCT05111613) has randomized patients to mechanical thrombectomy with the Inari FlowTriever or CDT, with the goal of comparing rates of all-cause mortality, intracranial bleeding, clinical deterioration or bailout, and ICU length of stay [66]. While this trial has completed enrollment, results have not yet been published.

## 8. High-Risk PE

**Figure 2:**
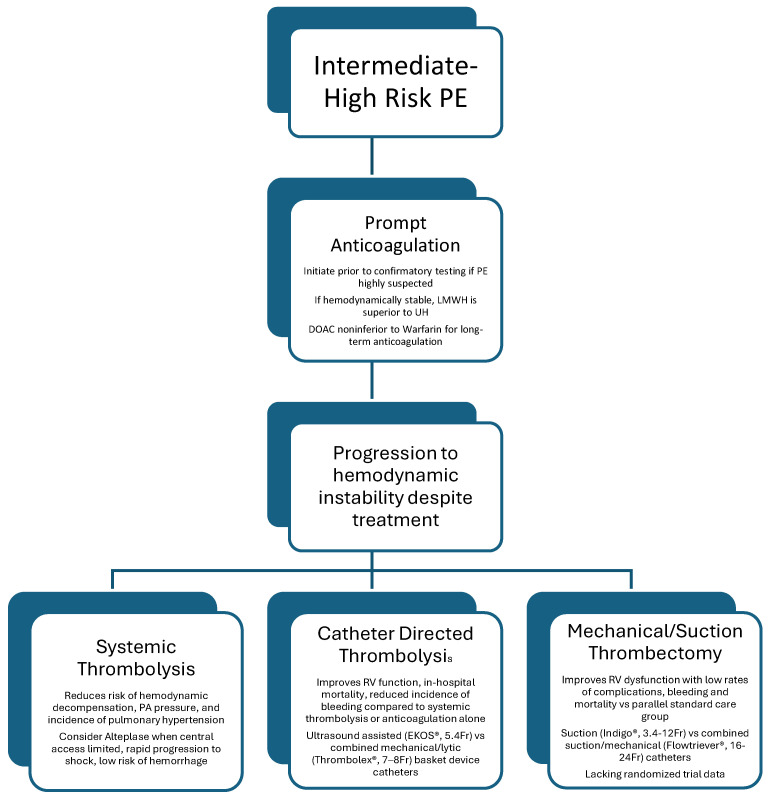
A summary of the evidence for the management of intermediate high-risk PE.

While comprising only 5% of pulmonary embolisms, high-risk PE confers a staggering mortality estimated at 30.2% at 30 days [2,69]. With high-risk patients being defined by sustained hypotension with BP < 90, vasopressor requirement, or presentation of cardiac arrest, this population remains understudied, with optimal management unclear in the face of advancing therapies. This section will cover treatment options and strategies for high-risk PE, including systemic thrombolysis, catheter-directed therapies, surgical embolectomy, vasopressor choice, and mechanical support, with a summary available in Figure 2.

### 8.1. Systemic Thrombolysis in High-Risk PE

RCT data that include IV systemic thrombolysis in hemodynamically unstable patients originates from five early studies, four of which that were conducted in the 1970s, and four that included 30 or fewer patients in the thrombolysis group. All five of these prospective trials utilized streptokinase or urokinase instead of tenecteplase as the thrombolytic agent [71,72,73,74,75]. Despite their small samples and heterogeneity, a meta-analysis of these trials with 2057 patients by Marti et al., comparing systemic thrombolysis followed by anticoagulation to anticoagulation alone, revealed a significant reduction in total mortality, PE-related mortality, and PE recurrence compared to anticoagulation alone, without a significant benefit in patients within the cohort who were hemodynamically stable [54]. Another meta-analysis of these trials by Wan et al. replicated these results, with a significant reduction in PE recurrence or death (9.4% in the thrombolytics plus anticoagulation group vs. 19.0% in the anticoagulation-alone group), with an NNT of 10 and no benefit demonstrated in trials including hemodynamically stable patients [76]. The benefit of systemic thrombolysis appears greatest when administered earlier in the illness course, but with residual benefit still observed 6 to 14 days after symptom onset [77]. These older studies provide the backbone of evidence for systemic thrombolysis. Notably, this therapy comes at the expense of a 9.9% rate of bleeding and 1.7% rate of intracranial hemorrhage [54].

### 8.2. CDT and MT in High-Risk PE

Data regarding catheter-directed outcomes specifically in high-risk PE patients are limited, with trials often combining intermediate high- and high-risk patients into a single cohort, such as in the SEATLE II trial. In this trial, decreases in RV/LV ratio between high-risk (*n* = 31) and non-high-risk (*n* = 119) subgroups were similar (−0.50 vs. −0.43, respectively), along with similar decreases in pulmonary artery systolic pressure. Massive PE patients were more likely to experience major bleeding compared to non-high-risk patients (23% vs. 7%) [58]. An analysis of the FLAME trial that included 53 high-risk PE patients undergoing MT with the FlowTriever system compared to a parallel group receiving systemic thrombolysis (68.9%) or anticoagulation alone (23%) demonstrated a lower rate of the primary outcome of all-cause mortality, bailout to another thrombus removal modality, clinical deterioration, or major bleeding (17% vs. 63.9%). More specifically, in-hospital mortality was lower in the MT arm compared to the comparator group (1.9% vs. 29.5%) [78]. A meta-analysis of 594 patients from both uncontrolled prospective and retrospective studies examining patients with massive PE that underwent CDT or MT demonstrated a rate of success (defined as successful hemodynamic stabilization, resolution of hypoxia, and survival to hospital discharge) of 86.5%, with a risk of major complications of 2.4% [79]. A majority of these patients underwent CDT or MT as their initial PE therapy without prior systemic IV thrombolysis. While likely an important area of future research, the overall body of evidence for upfront catheter therapies remains too small to replace systemic thrombolysis as the standard of care for high-risk PE.

### 8.3. Surgical Embolectomy in High-Risk PE

Surgical embolectomy is a reasonable approach for patients with high-risk PE who have failed thrombolysis or where thrombolysis is contraindicated. However, prospective randomized trial data comparing this approach to catheter-directed therapies or as first-line therapy are limited. Registry data of patients from 1995–2005 with high-risk PE who failed initial IV thrombolytic therapy were, at the discretion of the physician, directed to undergo surgical thrombectomy or repeat IV thrombolytic therapy. Although the surgical group included only 14 patients, this group demonstrated a lower rate of recurrent PE, bleeding complications, or PE related death, with a trend toward better mortality (10 deaths in the repeat IV thrombolysis groups vs. 1 death in the surgical group) [80]. A multicenter registry of 214 patients with both intermediate- and high-risk PE between 1998 and 2014 who underwent surgical embolectomy demonstrated a relatively low in-hospital mortality rate of 11.7%, with higher mortality in patients experiencing preoperative cardiac arrest [81]. More recent data from patients with both intermediate- and high-risk PE enrolled between 2011 and 2015 demonstrated an even lower mortality of 7%, with 100% survival in patients with intermediate-risk PE [82]. A large retrospective comparison of 2111 patients receiving surgical thrombectomy vs. IV thrombolysis as first-line therapy in the state of New York revealed no significant difference in 30-day mortality (15.2% in the IV thrombolytic group vs. 13.2% in the surgical group). However, an increased risk of stroke or reintervention for PE at 30 days and recurrence of PE requiring hospitalization at 5 years were observed in the thrombolysis arm [83].

While the data for surgical embolectomy appear promising, many of these studies utilize highly experienced and specialized centers (i.e., Massachusetts General Hospital, Brigham and Women’s Hospital, Kobe University Hospital, Mount Sinai Hospital) and suffer from selection biases, with no prospective randomized head-to-head comparisons with IV thrombolysis. In the largest nationwide inpatient sample of surgical thrombectomy outcomes amongst 2709 patients from 1050 participating institutions with varying levels of expertise, the overall inpatient mortality rate of 27.2% was higher than mortality seen in studies including only high-volume centers [84]. Discussions with PERT cardiovascular surgeons and an accounting of local expertise, thrombectomy volume, and the availability of resources, such as cardiac anesthesiology, perfusionists, ECMO circuits, and cardiac intensive care support staff, must all be considered prior to embarking on this treatment pathway. Thus, guideline recommendations continue to reserve the use of surgical embolectomy to patients who are unable to tolerate or are resistant to IV thrombolysis. However, additional consideration of this approach should be given to patients with thrombus found within the right-sided chambers of the heart, thrombus in transit, or thrombus overlying a patent foramen ovale, given that these situations often result in quick deterioration and mortality of up to 29% [85].

## 9. Adjunct Therapies

### 9.1. Volume Management

Management of fluids in the setting of pulmonary embolism should be individualized based on a patient’s volume status. In the DiPER trial, 276 patients with intermediate-risk PE were randomized to a single 80 mg IV dose of either furosemide or placebo. A significantly greater number of patients in the treatment group experienced improvements in PESI score characteristics, namely, tachycardia, low blood pressure, and hypoxia [86]. Importantly, 78.9% of patients in the treatment group had a BNP of >200 pg/mL, with an average NT proBNP of 2018 pg/mL. Conversely Mercat et. al. were able to demonstrate improvements in cardiac index after a 500 cc bolus of IV dextran in 13 patients with pulmonary embolism [87]. However, a negative correlation between right ventricular diastolic volume and improvement in cardiac index after fluids was demonstrated. A retrospective review of 70 consecutive patients with intermediate-risk PE who received either repeated boluses of furosemide (mean dose 78 mg) or resuscitation with fluids (mean volume 1.6 L) was also conducted, with similar baseline hemodynamic characteristics in each group. Patients receiving furosemide demonstrated a significant decrease in shock index and oxygen requirements, as well as an increase in blood pressure by 24 h, which was not demonstrated in the fluid resuscitation arm. Urine output directly correlated with RV dilation in the furosemide group. Finally, animal models using iatrogenic-induced pulmonary embolism in canines who were then given either 100 mL of IV dextran vs. norepinephrine suggest that volume expansion leads to increased rates of RV failure compared with vasopressor support alone [88]. While the ESC guidelines make a cautious recommendation for volume loading with ≤500 mL of IV fluids, they further encourage ultrasound and hemodynamically guided assessment of volume status to help guide management. The body of evidence would seem to favor a diuretic strategy over fluid resuscitation; however the benefit, seems to be greatest in those with the most profound RV dilation and dysfunction, thus necessitating a quality assessment of volume status and right- sided cardiac pressures.

### 9.2. Vasopressors in High-Risk PE

Evidence for the ideal vasopressor in patients with high-risk PE is lacking and derived from a mix of animal models and small human case series. Norepinephrine has been advocated by the ESC guidelines, with canine models demonstrating improvement in stroke volume and RV offloading without increasing pulmonary vascular resistance [89]. Dobutamine may also be beneficial in supporting RV function and cardiac output, with a small case series demonstrating improvement in oxygen delivery and peripheral oxygen saturation [90]. However, dobutamine may have the consequence of worsening ventilation–perfusion mismatching, as well as inducing systemic vasodilation and hypotension. The combination of norepinephrine and epinephrine has been demonstrated to be effective in patients with PE-associated shock refractory to dobutamine, with rapid reductions in pulmonary and right sided cardiac pressures while simultaneously increasing systemic arterial pressure [91]. However, these data come from a single case report. Additionally, isoproterenol has been studied via right heart catheterization in a small case series of nine patients with high-risk PE, demonstrating increases in heart rate, systemic arterial pressure, stroke volume, cardiac output, and PaO_2_, while decreasing pulmonary vascular resistance [92]. However, this therapy came at the expense of significant tachycardia. Use of these medications will depend on a thorough assessment of hemodynamic parameters, including RV function, pulmonary vascular resistance, degree of hypotension, and the patient’s individual comorbidities. Cardiology, pulmonology, and critical care specialists will need to collaborate to decide on the optimal regimen that supports hemodynamic stability.

### 9.3. Extracorporeal Membrane Oxygenation

The use of extracorporeal membrane oxygenation (ECMO) has demonstrated efficacy in case studies involving high-risk PE; however, randomized prospective data with suitable control groups are lacking. Older cohort data from 21 patients with massive PE cannulated for extracorporeal life support (ECLS) during 1992–2005 revealed in-hospital mortality of 38% [93]: 57% of these patients received ECLS after failing initial treatment with systemic thrombolysis or suction thrombectomy, and 19% underwent surgical thrombectomy. Later data acquired from ECMO patients between 2000 and 2011 demonstrated a much lower 12.5% in-hospital mortality rate, perhaps reflecting evolving PE care and improvements in extracorporeal support gained over time. This lower mortality occurred despite the enrollment of a relatively sick patient population, including patients who were either in cardiogenic shock, failed thrombolysis, or were not candidates for thrombolytic therapy—46% of these patients presented with cardiac arrest [94]. The most recent outcomes published in a systematic review of patient-level data derived from case series and case studies during 2006–2019, including 128 participants with massive PE. ECMO use was associated with in-hospital mortality of 22%, with definitive therapy deployed via CDT, MT, and surgical thrombectomy. Overall, 85.1% were able to be weaned off of ECMO, with the most common post-extracorporeal life support complications being bleeding (23.4%) and renal failure (8.6%) [95]. In sum, these data suggest gradually improving outcomes associated with ECMO use in PE over the years.

Considering 30-day mortality amongst all cardiogenic shock patients receiving ECMO can approach 50% [96], and high-risk PE mortality up to 65% in those requiring CPR, survival data for ECMO as a bridge to other advanced therapy appear relatively promising [11]. While ECMO does not address the underlying pathology of the disease and should only be utilized as a means to maintain hemodynamic stability and perfusion while definitive therapies with thrombolysis or thrombectomy are pursued, it remains a valuable tool during the late-stage spiral of PE. The insight of cardiothoracic surgeons, critical care intensivists, and cardiologists can provide valuable advice on procedural considerations related to ECMO access, the hemodynamic, hematologic, and infectious risks associated with ECMO, and evaluation for the need of LV offloading to maintain optimal intracardiac pressures.

### 9.4. Right Ventricular Assist Devices

Right ventricular assist devices (RVADs) are another recently emerging support modality with therapeutic potential in patients who present with acute PE; however, utilization and published data remain scant, with a lack of any large retrospective cohorts in the literature and absent regulatory body approval. Devices include the Impella RP micro-axial flow pump. From a cohort of patients who developed acute RV failure and cardiogenic shock due to high-risk pulmonary embolism refractory to systemic thrombolysis and inotropic support, Elder et al. published results from a case series of five patients that received Impella support in addition to ultrasound-facilitated catheter-directed thrombolysis with the EKOS system [97]. These patients had a mean pulmonary artery pulsatility index of 1.6 despite marked RV dilation and dysfunction on echocardiography. They also had preserved LVEF > 50% in all cases, mean pulmonary artery pressure of 43, and a cardiac index of 1.69 L/min/m^2^ prior to RVAD insertion. Mean time to Impella RP insertion was 3.2 days. An improvement in mean cardiac output was demonstrated, to 2.5 L/min/m^2^, along with an improvement in TAPSE in all patients prior to RVAD removal. The average duration of Impella support was 3.8 days. All five patients survived to hospital discharge and were alive at 1 month follow-up. Taha et al. published additional case reports that replicated similar clinical success with the Impella RP system following catheter thrombolysis, enabling rapid weaning off inotropic support [98].

Other devices, such as the left atrial–femoral artery bypass system Tandem Heart, have enabled effective treatment of cardiogenic shock refractory to systemic thrombolysis [99]. The ProtekDuo device, which allows blood to bypass the right ventricle via a right atrial inlet canula and flow out to the pulmonary arteries, might provide hemodynamic support and enable recovery of PE-induced RV dysfunction after definitive thrombolysis or thrombectomy. However, data to support this approach aew lacking [100]. In a systematic review of mostly RVAD case reports in PE, all 17 patients included were successfully weaned from RVAD support, with a mean duration of 3.9 days of support, and an overall survival to hospital discharge of 94%. Patients were relatively young, with an average age of 48 years, with utilization of a broad array of other devices including the Medtronic Inc. BioMedicus brand of centrifugal pumps, the Sarns system by 3M, BVS system by Abiomed, and the CentriMag system by Abbot [101]. However, the choice of RVAD will depend not only on careful consideration and balancing of the vascular access requirements, hemodynamic effects, and hematologic consequences related to each device but also the individual experience and familiarity of the interventional cardiologists and cardiothoracic surgeons involved.

### 9.5. Pulmonary Embolism Response Teams

Utilization of a multidisciplinary consultation team of cardiology (general and interventional), pulmonology, intensive care, vascular surgery, vascular medicine, hematology, emergency medicine, interventional radiology, hematology and cardiothoracic surgery specialists may be useful in cases of intermediate- and high-risk PE cases. Early retrospective data on 77 patients treated by a PERT program at the University of Kentucky Medical Center suggested a decrease in both ICU length of stay and the overall duration of hospitalization [102]. The team was composed of specialists from pulmonary critical care, interventional radiology, cardiac surgery, cardiology, and vascular surgery, but also included emergency medicine, hospital medicine, nursing, and pharmacy specialists, and enabled CDT to be employed in 61.8% of intermediate high-risk cases. The involvement of a broad array of specialties in this study is especially important in considering international hospitals, where expertise in thrombosis may come from disciplines such as internal medicine, not typically associated with advanced PE care in the United States. Furthermore, incorporation of non-physician members may allow for more comprehensive care, including knowledge about appropriate dosing adjustments, administration of medications and the more granular aspects of bedside patient care and monitoring. Further data from the implementation of a PERT at the Cleveland Clinic showed an 8.7% absolute decrease in major bleeding, a 3.7 h decrease in time to therapeutic anticoagulation, decreased use of inferior vena cava filters, and a significant 3.8% absolute 30-day/inpatient rate of mortality [103]. This study also demonstrated an increased use of thrombolytic and catheter-based therapies, with differences most pronounced in patients with intermediate- and high-risk PE. Support for PERT includes international cohorts, including a 3-year German study of 88 patients that showed an associated 9.1% reduction in PE-related mortality and 1.1% reduction in all-cause mortality [104].

However, the efficacy of PERTs has varied by institution, with the published experience at the University of Michigan demonstrating a decrease in utilization of advanced therapies and no difference in mortality when compared to the pre-PERT group [105]. Possible reasons cited for the lack of benefit were unclear baseline utilization of CDT prior to the implementation of a PERT, and that perhaps the PERT allowed for more judicious allocation of CDT resources, potentially by reducing its use in poorly indicated candidates. Beth Israel Deaconess Medical Center similarly analyzed data pre- and post-PERT implementation. While no significant differences were identified in mortality or bleeding, an increase in ordering of risk-stratification measurements (cardiac biomarkers, echocardiograms, etc.) and a decrease in placement of IVC filters was demonstrated [27].

A meta-analysis of 22 studies demonstrated decreased in-hospital stay by a mean of 1.6 days and increased utilization of advanced therapies with PERT implementation (RR 2.67, 95% CI 1.29–5.50) . While overall mortality was not significantly different, a trend toward benefit was demonstrated particularly in intermediate- and high-risk patients (RR 0.71, 95% CI 0.45–1.12) [106].

Furthermore, the educational benefit of PERT has been demonstrated to improve the confidence and knowledge of residents and fellows in diagnosing and managing intermediate- and high-risk PE, particularly with surgical embolectomy and thrombolysis, compared to pre-PERT cohorts, with trainees surveyed largely in favor of the continued implementation of PERT [107]. This educational benefit has been shown to extend to hospital faculty and house staff as well, with demonstrated improvements in ability to risk-stratify patients accurately and increased comfort in managing PE [108]. The ESC guidelines support the use of a PERT as a class IIa recommendation (Table 2).

### 9.6. Future Directions

**Figure 3:**
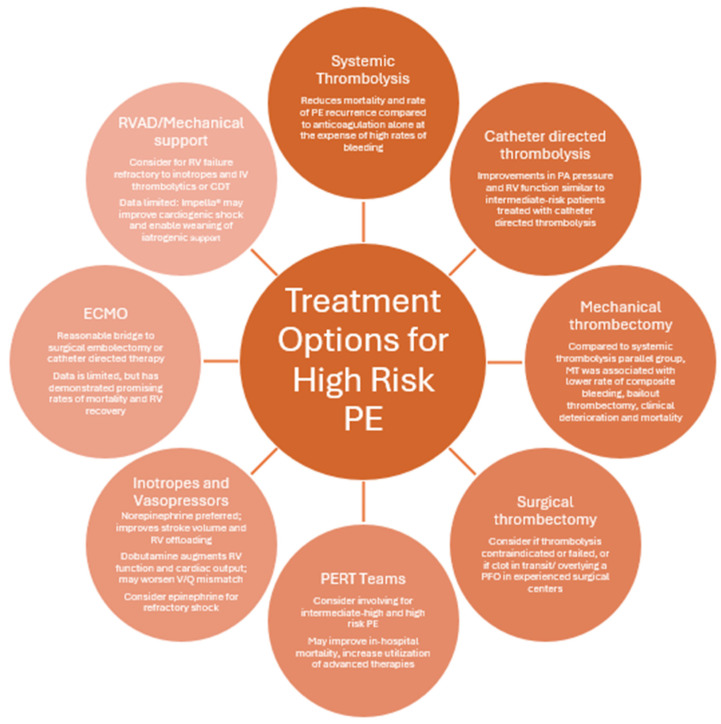
A summary of the evidence for the management of high-risk PE.

Areas for future development include evaluating new modalities and technologies within the CDT field, comparing outcomes between CDT devices, randomized head-to-head studies comparing systemic thrombolysis/anticoagulation to CDT, and identification of optimal first-line options for intermediate high- and high-risk patients, particularly for those who either fail or have contraindications to systemic thrombolysis (Figure 3). Parsing out the intermediate high-risk category to preemptively identify patients expected to require more than anticoagulation alone and identifying the long-term effects of CDT compared to anticoagulation on long term outcomes requires further inquiry.

There are several randomized controlled trials currently enrolling intermediate-risk participants to study the effects of combined CDT and anticoagulation compared to anticoagulation alone. STORM-PE is a prospective multicenter randomized controlled trial utilizing the Indigo catheter to compare the efficacy of aspiration thrombectomy plus anticoagulation versus anticoagulation alone [115]. Targeting an enrollment of 100 patients, the primary outcome focuses on examining very early indicators of treatment response, measuring change in RV/LV ratio at 48 h.

The HI-PEITHOS randomized controlled trial will instead focus not only on the modality of ultrasound-assisted thrombolysis combined with anticoagulation, compared to anticoagulation alone. but also examine outcomes extended to 7 days from randomization [62]. These outcomes include 7-day mortality, cardiopulmonary decompensation, and recurrence. The study plans to enroll 406 patients from a multinational cohort of institutions, and will be industry-sponsored.

As described above, PEERLESS II is an industry-sponsored, multicenter, randomized controlled trial enrolling up to 1200 patients and including up to 100 sites examining the efficacy of mechanical thrombectomy using the FlowTriever system compared to anticoagulation alone on a hierarchical composite win ratio consisting of overall mortality, clinical deterioration, or hospital readmission over a longer period of 30 days [66]. Outcomes will also be measured at 90 days, with the additional outcomes of major bleeding, quality of life, and functional status also recorded.

**Figure 4:**
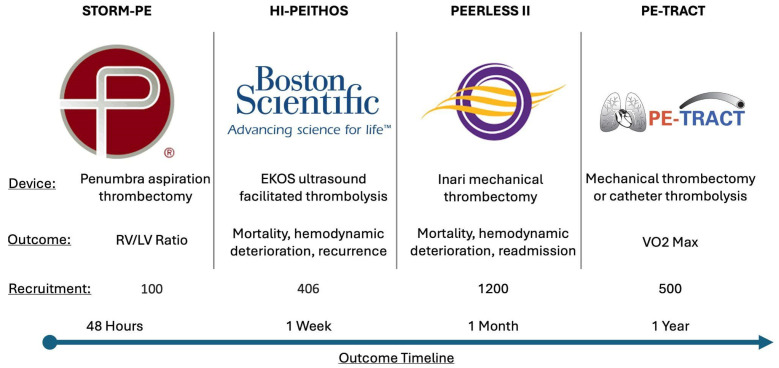
Trials examining the effects of CDT and anticoagulation against anticoagulation alone that are actively enrolling.

Results of the ELOPE study, which recruited 100 patients diagnosed with pulmonary embolism treated with primarily anticoagulation, showed that 46% of participants had significantly reduced percentage-predicted VO_2_ max on cardiopulmonary exercise testing even 1 year post-diagnosis [116]. This may be in part due to residual pulmonary vascular obstruction left over and inadequately dismantled in up to 59% of patients treated with anticoagulation alone. This is in contrast to catheter therapies such as MT, which may reduce the incidence of residual obstruction [117]. PE-TRACT, a randomized, multicenter, open label, assessor-blinded controlled trial, intends to evaluate, without industry sponsorship, whether the addition of CDT to anticoagulation in patients with intermediate high-risk PE might improve long-term functional status and exercise capacity when compared to anticoagulation alone [118] (Figure 4).

## 10. Conclusions

Since the introduction of CT angiography, the detection of pulmonary embolism has improved substantially, with an 80% increase demonstrated between 1998 and 2006 [119]. While there is likely a component of over-detection, the ability to diagnose PE in timely fashion allows for earlier intervention. In addition to detection, advances in CDT, MT, and surgical embolectomy have expanded the armament of treatments available for higher-severity pulmonary emboli. As of yet, newer therapies have not shown superiority with level I evidence to the current standards of care, including anticoagulation for intermediate high-risk and thrombolysis for high-risk patients. While low-risk patients clearly benefit from anticoagulation alone, with a single-drug DOAC strategy that can facilitate early discharge and even outpatient management in appropriately risk-stratified patients, in intermediate high-risk PE, the preferred treatment for patients who fail anticoagulation or progress to clinical instability remains to be defined.

Between systemic anticoagulation and catheter delivered therapies, data appear especially promising for MT in terms of avoiding deterioration, reducing time in the ICU, and maintaining low rates of intracranial and major bleeding. Given the heterogeneity of this population, preemptively identifying the large proportion of patients within this group who are likely to deteriorate on standard therapies alone remains pivotal. Further advancements in risk stratification to consistently identify these patients is a necessary step to discern patients most likely to benefit from advanced therapies. PERTs may be most beneficial amongst this population, and while a mortality difference has yet to be determined, benefits including closer collaboration between specialties, enhanced educational opportunities for both trainees and faculty, and optimizing appropriate usage of advanced therapies make them a vital component of PE management.

Amongst high-risk patients, reperfusion remains the cornerstone of treatment; however, choosing when and how to employ CDT, systemic thrombolysis, and surgery requires not only careful consideration of institutional expertise, patient comorbidities, and hemodynamics but also needs further research, as the optimal upfront strategy remains unclear. Stabilization with ECMO and ventricular assist devices can be helpful and is often necessary as a bridge to treatment. Initial survival data with these devices appear promising; however, given the critical nature of patients using them, this population remains largely unstudied and their role in management has yet to be firmly established.

Understanding the methodologic limitations that underscore the current published evidence behind emerging therapies is critical in deciding which modalities should be ultimately adopted, particularly when guideline recommendations remain imprecise. Despite huge technological advancements, unbiased and conclusive data remain insufficient. Until results from ongoing and future trials become available, multidisciplinary collaboration and frequent reevaluation of the literature should remain cornerstones in the evolving treatment of acute pulmonary embolism (Table 3).

## Figures and Tables

**Table 1:** A comparison of scoring categories for several clinical risk calculators.

	PESI	sPESI	Bova	Hestia	FAST	CPES
Lower Risk	Class 1–2	≤1	0–2	0	<3	<4
Elevated Risk	Intermediate: Class 3 High: Class 4–5	2–6	Intermediate: 3–4 High: 5–7	1–11	3–5	4–6

**Table 2:** Comparison of AHA, ESC, CHEST, and NICE guidelines for acute PE.

	AHA (2011, 2019, 2023 Scientific Statements) [10,109,110]	ESC [9]	CHEST [111]	NICE [112,113,114]
High-risk PE: CDT/surgery	Depending on local expertise, either catheter embolectomy and fragmentation or surgical embolectomy is reasonable for patients with massive PE and contraindications to fibrinolysis (class IIa; Level of Evidence C). Catheter embolectomy and fragmentation or surgical embolectomy is reasonable for patients with massive PE who remain unstable after receiving fibrinolysis (class IIa; level of evidence C). Observational data support a role for active thrombus removal in patients with thrombus in transit, although this decision is also influenced by the size and nature of the thrombus and the severity of the initial PE.	Surgical pulmonary embolectomy is recommended for patients with high-risk PE in whom thrombolysis is contraindicated or has failed.	In patients with acute PE associated with hypotension and who have (i) a high bleeding risk, (ii) failed systemic thrombolysis, or (iii) shock that is likely to cause death before systemic thrombolysis can take effect (e.g., within hours), if appropriate expertise and resources are available, we suggest catheter-assisted thrombus removal over no such intervention (grade 2C).	Percutaneous thrombectomy for massive pulmonary embolism (PE) should be used only in research. Percutaneous thrombectomy is usually used if someone has had a massive PE and they cannot have surgery, and when thrombolysis is contraindicated or has failed. Systemic thrombolysis may be used for massive or submassive PE and rarely open surgical embolectomy.
High-risk PE: systemic thrombolysis	Fibrinolysis is reasonable for patients with massive acute PE and acceptable risk of bleeding complications (class IIa; level of evidence B).	Systemic thrombolytic therapy is recommended for high-risk PE	Fibrinolysis may be considered for patients with submassive acute PE judged to have clinical evidence of adverse prognosis (new hemodynamic instability, worsening respiratory insufficiency, severe RV dysfunction, or major myocardial necrosis) and low risk of bleeding complications (class IIb; level of evidence C).	Consider pharmacological systemic thrombolytic therapy for people with PE and hemodynamic instability.
Intermediate-risk PE: CDT/surgery	Either catheter embolectomy or surgical embolectomy may be considered for patients with submassive acute PE judged to have clinical evidence of adverse prognosis (new hemodynamic instability, worsening respiratory failure, severe RV dysfunction, or major myocardial necrosis) (class IIb; level of evidence C). Among patients with intermediate-risk PE, a careful assessment for factors that elevate risk of decompensation should be undertaken, including elevated PESI or simplified PESI score, severe PE-related functional impairment, and objective signs of severely diminished end-organ perfusion or stroke volume. In those who meet these criteria and have non-prohibitive bleeding risk, systemic thrombolysis or CDL may be considered. Catheter-based embolectomy represents an option for patients in this cohort with elevated bleeding risk, with the caveat that concerns for procedural hemodynamic or respiratory decompensation exist with these technologies.	As an alternative to rescue thrombolytic therapy, surgical embolectomy or percutaneous catheter-directed treatment should be considered for patients with hemodynamic deterioration on anticoagulation treatment (2A)	In patients with acute PE associated with hypotension and who have (i) a high bleeding risk, (ii) failed systemic thrombolysis, or (iii) shock that is likely to cause death before systemic thrombolysis can take effect (e.g., within hours), if appropriate expertise and resources are available, we suggest catheter-assisted thrombus removal over no such intervention (grade 2C).	Systemic thrombolysis may be used for massive or submassive PE and, rarely, open surgical embolectomy.
Intermediate-risk PE: systemic thrombolysis	Fibrinolysis may be considered for patients with submassive acute PE judged to have clinical evidence of adverse prognosis (new hemodynamic instability, worsening respiratory insufficiency, severe RV dysfunction, or major myocardial necrosis) and low risk of bleeding complications (class IIb; level of evidence C).	Rescue thrombolytic therapy is recommended for patients with hemodynamic deterioration on anticoagulation treatment	Fibrinolysis may be considered for patients with submassive acute PE judged to have clinical evidence of adverse prognosis (new hemodynamic instability, worsening respiratory insufficiency, severe RV dysfunction, or major myocardial necrosis) and low risk of bleeding complications (class IIb; level of evidence C).	Systemic thrombolysis may be used for massive or submassive PE and, rarely, open surgical embolectomy.
Risk-stratification and scoring systems	PESI and sPESI should be used to identify patients with low 30-day all-cause mortality who are suitable for outpatient management.			
	Acute PE should be classified as low, intermediate, and high risk	Acute PE should be categorization into low, intermediate low, intermediate high, and high risk. Utility of Bova score in guiding management is unclear.		
Timing of AC	Therapeutic anticoagulation during the diagnostic workup should be given to patients with intermediate or high clinical probability of PE and no contraindications to anticoagulation (class I; level of evidence C).	Initiation of anticoagulation is recommended without delay in patients with high or intermediate clinical probability of PE, while diagnostic workup is in progress (grade 1A).	In patients with a high clinical suspicion of acute PE, we suggest treatment with parenteral anticoagulants compared with no treatment while awaiting the results of diagnostic tests (grade 2C).	Interim therapeutic anticoagulation while awaiting CTPA.
Type of AC	Therapeutic anticoagulation with subcutaneous LMWH, intravenous or subcutaneous UFH with monitoring, unmonitored weight-based subcutaneous UFH, or subcutaneous fondaparinux should be given to patients with objectively confirmed PE and no contraindications to anticoagulation (class I; level of evidence A).	If anticoagulation is initially parenterally, LMWH or fondaparinux is recommended (over UFH) for most patients.	In patients with a high clinical suspicion of acute PE, we suggest treatment with parenteral anticoagulants compared with no treatment while awaiting the results of diagnostic tests (grade 2C).	Do not routinely offer unfractionated heparin (UFH) with a VKA to treat confirmed proximal DVT or PE unless the person has renal impairment or established renal failure (see recommendations 1.3.13 and 1.3.14) or an increased risk of bleeding. For people with confirmed PE and hemodynamic instability, offer continuous UFH infusion and consider thrombolytic therapy. Offer either apixaban or rivaroxaban to people with confirmed proximal DVT or PE
PERT	PERT may serve as a future platform for prospective observational and experimental research into technologies involved in the management of PE. It is unclear whether the PERT framework for acute PE care improves patient outcomes and is cost-effective. Formal health systems evaluation and implementation research on PERT have not yet been performed.	Setup of a multidisciplinary team and a program for the management of high- and (in selected cases) intermediate-risk PE should be considered, depending on the resources and expertise available in each hospital (2A).		[For anticoagulation in patients with renal impairment] The committee emphasized the importance of following the SPCs and locally agreed protocols, and seeking advice from specialist colleagues or a multidisciplinary team to ensure correct dosing and monitoring.
VA-ECMO	Relative to the severity of illness at presentation, survival and RV recovery are excellent. Analysis of the impact of the heterogeneity of treatment patterns and clinical presentation of patients is needed to better risk-stratify patients and to determine comparative treatment modality efficacy.	ECMO may be considered in combination with surgical embolectomy or catheter-directed treatment in refractory circulatory collapse or cardiac arrest.		
RVADs	Emerging percutaneous RV MCS with or without an associated oxygenator can be used to support a failing RV. Data on their efficacy in the setting of PE are limited.			

**Table 3:** Summary of available literature regarding acute PE management.

Low risk	Therapeutic anticoagulation: Hestia study (2011): Lower risk of VTE recurrence and death at 3 months when treated with anticoagulation in the outpatient setting OTPE (2011): Outpatient LMWH is noninferior to inpatient treatment in terms of recurrence, bleeding and death Home PE (2021): HEISTA score was equal to sPESI for triaging patients suitable for outpatient treatment EINSTEIN (2012): Rivaroxaban with short-term noninferiority and long-term superiority to warfarin AMPLIFY (2013): Apixaban noninferior to warfarin in preventing VTE recurrence or death HOKUSAI (2013): Edoxaban noninferior to warfarin in preventing recurrence of symptomatic VTE
Intermediate low risk	Anticoagulation remains the preferred treatment. Catheter-directed thrombolysis: Large scale data still required Subgroup analysis from the SUNSET (2021) and RESCUE (2022) may suggest benefit
Intermediate high risk	Anticoagulation remains the preferred treatment. Despite subgroup analysis of trials with promising results, data are insufficient to recommend alternative treatments as first line. Systemic thrombolysis: PAIMS 2 (1992), MOPETT (2013), and PEITHO (2014) trials suggest benefit in lowering pulmonary artery pressure in patients who develop hemodynamic instability Catheter-directed thrombolysis: ULTIMA (2013): Found improvement in RV/LV ratio for the EKOS catheter 24 h after treatment SEATLE II (2015): Found that improvements in RV/LV ratio persisted to 48 h after treatment, with <1% rate of bleeding RESCUE (2022): Evaluated with BASHIR catheter with improvements in RV/LV ratio at 48 h after treatment and <1% rate of bleeding CANARY (2022): Improvements in RV/LV ratio from CDT persisted to 3 months posttreatment Meta-analysis data found CDT was associated with lower rates of mortality and major bleeding compared to systemic thrombolysis and lower mortality compared to anticoagulation alone; however, no RCT data exist to confirm this Mechanical thrombectomy and catheter-directed suction EXTRACT-PE (2021) and FLAME (2023): Evaluated suction thrombectomy and FlowRetriever systems, demonstrating improvements in RV/LV ratio, low mortality, and lower rates of bleeding compared to systemic thrombolytics FLASH registry (2022): Ongoing study recently completing enrolment for MT, with preliminary data showing 6-month mortality of 4.6% and RV/LV ratio reduction of 35% on average PEERLESS (2024): RCT demonstrating superiority of mechanical thrombectomy over catheter thrombolysis, with reduced incidence of clinical deterioration, ICU utilization, and hospital readmission Volume management: Ideally should be individualized. DiPER trial (2022) and several retrospective reviews found furosemide improved RV offloading and cardiac index in patients with severe RV dysfunction.
High-risk PE	Systemic thrombolytics RCT data come from five trials in the 1970s showing improvement in mortality and PE recurrence for hemodynamically unstable patients with rates of major bleeding range of 9.9–19.2% Mechanical thrombectomy FLAME subgroup (2023): Compared to a parallel group receiving systemic thrombolysis, MT was associated with a reduced incidence of a composite of all-cause mortality, bailout to another thrombus removal modality, clinical deterioration, and major bleeding (17% vs. 63.9%) Catheter-directed thrombolysis SEATLE II subgroup: Reduction in RV/LV ratio between massive and submassive subgroups were similar (−0.50 vs. −0.43, respectively) Ideal vasopressor Data limited to human and animal case series Norepinephrine found to improve stroke volume and RV offloading without increasing pulmonary vascular resistance Dobutamine may improve RV function and cardiac output, but may worsen V/Q mismatch and hypotension Norepinephrine and epinephrine have some evidence for treating shock refractory to dobutamine PERTs Data from individual institutions vary in terms of mortality, IVC filter placement, and rates of bleeding Meta-analysis data suggest reduction in hospital stay without affecting mortality for intermediate-/high-risk patients Surgical thrombectomy Comparative data between surgical thrombectomy and catheter-directed treatments of IV thrombolytics are limited Meta-analysis data showed an overall mortality of 27.2%, although this may vary between institutions with more experience ECMO Case series studies found promising rates of mortality; however, data are limited RVAD Small case series studies suggest utility in treating cardiogenic shock refractory to systemic thrombolysis or inotropic support
